# Thyroid nodules in children and adolescents: Investigation and management

**DOI:** 10.1111/jpc.16257

**Published:** 2022-11-16

**Authors:** Jessica L Sandy, Angela Titmuss, Shihab Hameed, Yoon Hi Cho, Gideon Sandler, Paul Benitez‐Aguirre

**Affiliations:** ^1^ Institute of Endocrinology and Diabetes The Children's Hospital at Westmead Westmead New South Wales Australia; ^2^ Child & Adolescent Health Faculty of Medicine and Health, University of Sydney Sydney New South Wales Australia; ^3^ Division of Women Children and Youth, Royal Darwin Hospital Darwin Australia; ^4^ Menzies School of Health Research Charles Darwin University Darwin Australia; ^5^ Paediatric Endocrinology Sydney Children's Hospital Network New South Wales Australia; ^6^ School of Women and Children University of New South Wales New South Wales Australia; ^7^ Department of Surgery The Children's Hospital at Westmead Westmead New South Wales Australia; ^8^ School of Health Sciences University of Sydney New South Wales Australia; ^9^ Present address: Institute of Diabetes and Endocrinology, The Children's Hospital at Westmead Corner Hawkesbury Road and, Hainsworth St Westmead NSW 2145 Australia

**Keywords:** paediatrics, thyroid cancer, thyroid nodules, thyroidectomy

## Abstract

Clinically detectable thyroid nodules are less common in children than adults. However, they are associated with an increased risk of malignancy. Therefore, thorough evaluation of paediatric thyroid nodules is necessary, and an understanding of the features associated with a higher risk of malignancy is important to guide management and referral. Thyroid cancer in children differs significantly from that seen in adults in terms of genetics, presentation, response to treatment and prognosis. Children often present with more advanced disease, but the vast majority have excellent long‐term prognosis. Evaluation and management of thyroid nodules and thyroid cancer require a multidisciplinary team approach and involvement of specialists with experience in this field. This review summarises investigative pathways for thyroid nodules in children and outlines current management strategies for paediatric thyroid nodules and cancer.

**Key Points**

1. Solitary thyroid nodules in children are more likely to be malignant compared with nodules in adults.
2. Thyroid cancer is more frequently metastatic in children than in adults.
3. Thyroid cancer in children has an excellent long‐term prognosis.
4. Risk factors for paediatric thyroid cancer include radiation exposure, family history (particularly for medullary thyroid carcinoma), and the presence of autoimmune thyroiditis.
5. Adequate evaluation and management of paediatric thyroid nodules and paediatric thyroid cancer require a multidisciplinary team approach.
6. Management of paediatric thyroid cancer should be within a multidisciplinary team involving a paediatric endocrinologist, high‐volume thyroid surgeon, nuclear medicine physician, paediatric radiologist, histopathologist, paediatric oncologist and appropriate psychosocial support.

## Thyroid Nodules in Children

The estimated prevalence of solid thyroid nodules in children is 1–1.7%,^1^, ^2^ with incidence increasing with age. Management of thyroid nodules is a common challenge encountered by paediatricians, with thyroid abnormalities, including nodules or cystic lesions, seen in 20–57% of children having ultrasounds of the neck for various indications.^2^, ^3^ The incidence of malignancy in a solitary paediatric thyroid nodule is between 19 and 26.4%,^4^, ^5^ higher than the 5% incidence seen in adults. Therefore, a familiarity with evaluating thyroid nodules in children is necessary in paediatric practice.

Differential diagnoses of thyroid nodules include structural, developmental and non‐neoplastic lesions, benign neoplasms and malignant neoplasms (Table 1). Importantly, many non‐neoplastic thyroid nodules are transient in children, and may reduce in size over time.^2^, ^6^ It is important to note that nodules in the thyroid upper poles are frequently impalpable even when large.^7^ Non‐palpable nodules detected incidentally by imaging have a relatively low malignancy rate (4%),^8^ and the American Thyroid Association (ATA) 2015 guidelines^1^ refrain from any recommendation regarding ultrasound use as a screening tool due to inconclusive evidence that early diagnosis of clinically undetectable lesions impacts the outcome.^1^

**Table 1:** Classification of thyroid nodules

Non‐neoplastic lesions	Nodule within autoimmune thyroiditis	Nodules develop in context of firm diffuse goitre.
	Colloid cyst	Benign, cystic nodule containing a central plug of avascular colloid.
	Multi‐nodular goitre	May have a dominant nodule within this, which has same malignancy risk as a solitary nodule; may occur in the context of genetic syndromes.
	Hyperplastic nodule	Polyclonal in origin (compared to monoclonal solitary nodules).
	Thyroglossal cyst	Developmental anomaly of thyroid gland.
		Located in midline between base of tongue and hyoid bone.
Benign neoplasms	Toxic adenoma	Have associated biochemical ± clinical hyperthyroidism.
	Non‐functioning follicular adenoma	Encapsulated, uniform, follicular cell differentiation.
Malignant neoplasms	Primary carcinoma (papillary/follicular/medullary/anaplastic)	May have associated cervical lymphadenopathy. Undifferentiated thyroid cancer is rare in children, papillary carcinoma is most common.
	Lymphoma	
	Metastasis from another site	

## Clinical Approach to a Child with a Thyroid Nodule

### Risk factors for malignancy

A clinical history should involve an assessment for risk factors for malignancy, including radiation exposure, chemotherapy, family history (particularly for medullary thyroid carcinoma (MTC)), autoimmune thyroiditis and endemic iodine deficiency (for follicular carcinoma).

The risk of thyroid cancer following thyroid irradiation is highest in those exposed at a younger age and in those who received a lower/scatter dose of 20–29 Gy. Radiation doses >30 Gy confer a lower risk, possibly because higher doses induce cell death rather than somatic mutations.^9^ There is often a long latency period; a review of over 1000 cases of thyroid cancer after childhood thyroid irradiation showed a mean time between exposure and cancer diagnosis of 28.9 years, with a low number of thyroid cancers developing within 5 years.^10^ Children's Oncology Group (COG) Guidelines recommend surveillance begin 5 years following radiation exposure with annual physical examinations.^11^ The additional use of screening ultrasounds in this high‐risk cohort is variable across centres and the literature.^1^, ^12^ Chemotherapy is also associated with a fourfold increase in risk for developing thyroid cancer and is additive to the risk of radiotherapy.^10^

Thyroid tumours may be seen in certain syndromes, such as the Carney complex, familial adenomatous polyposis, and Cowden, *DICER1*, Werner, McCune Albright and Li‐Fraumeni syndromes.^1^ A family history of MTC, parathyroid adenoma or phaeochromocytoma may suggest multiple endocrine neoplasia type 2 (MEN2) syndromes.^13^

Adult studies have demonstrated a clear association between autoimmune thyroiditis (AT, or Hashimoto's thyroiditis) and thyroid cancer, especially papillary thyroid cancer (PTC), with incidence in this population varying widely between studies (1.1–40.1%). Thyroiditis‐associated differentiated thyroid cancer (DTC) has a lower risk of extrathyroidal extension, lymph node metastases and tumour recurrence with an overall better prognosis.^14^ Paediatric studies also suggest an association between AT and PTC, with AT seen in 6.3–40% of children with PTC.^15^ A recent meta‐analysis showed that the incidence of PTC in children with AT varied between 0.7 and 7.8%, with higher incidence in those with positive thyroid antibodies and higher TSH levels.^16^ Current guidelines suggest that an ultrasound be performed in children with AT if there is a palpable nodule, gland asymmetry, or cervical lymphadenopathy on examination.^1^ There is, however, variability in practice with some physicians performing routine ultrasounds in all patients with AT.

### Examination

Physical examination includes assessment of whether the nodule is solitary or multiple and for the presence of cervical lymphadenopathy. It is important to evaluate for any signs of hyperthyroidism or local compression. A diffuse increase in size of a lobe or the entire thyroid gland, especially if associated with cervical lymphadenopathy, should prompt imaging given the risk of diffuse sclerosing form of PTC in children.^1^ Examination findings suggestive of malignancy include a clinically palpable nodule, larger nodule size, thyroid asymmetry, firm consistency, presence of cervical lymphadenopathy and increasing nodule size.^7^

### Investigations, management and indications for specialist referral

**Fig. 1:**
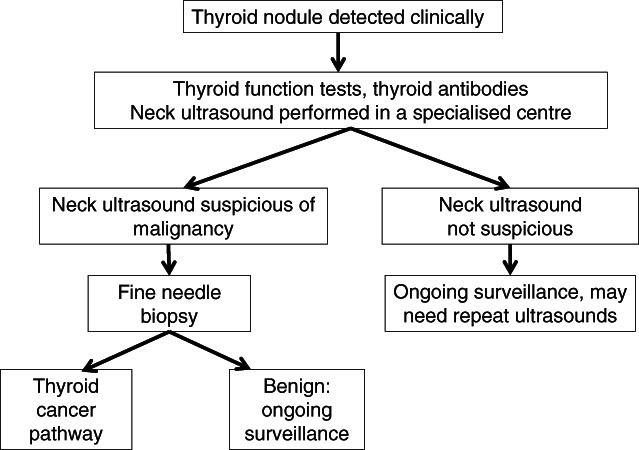
Suggested diagnostic pathway for investigation of thyroid nodules.

Figure 1 outlines a suggested diagnostic pathway for thyroid nodules in children. Investigations include thyroid function tests and anti‐thyroid antibodies (anti‐thyroglobulin and thyroid peroxidase antibodies). Hyperthyroidism with suppressed TSH suggests a toxic nodule.^17^, ^18^ Serum calcitonin levels should be measured if MEN2 is suspected as medullary cells produce calcitonin. A high serum level may suggest medullary thyroid cancer. The discovery of a medullary thyroid cancer should prompt investigations for phaeochromocytoma prior to any operative thyroid intervention.

Evaluation and management of thyroid nodules in children require a staged approach. Suspicious nodules should be reviewed in centres with paediatric thyroid expertise. Where there is uncertainty regarding further investigation, the authors recommend that this be done after multidisciplinary review which may require referral to a tertiary centre or an appropriate specialist (surgeon, endocrinologist and/or radiologist) with experience in managing paediatric thyroid nodules.

### Imaging of thyroid nodules

Ultrasound is the first‐line imaging investigation for thyroid nodules, and is used to assess the need for further evaluation and the timing thereof, including fine need aspiration biopsy (FNAB) and/or surgical resection. In addition, a ^99m^Tc pertechnetate diagnostic scan is suggested in cases of biochemical hyperthyroidism to delineate the underlying cause (e.g. a toxic nodule which is a rare occurrence in childhood).^7^

Given significant inter‐operator variability, ultrasounds should be performed and reported by technicians and radiologists who are familiar with paediatric thyroid nodules. Similarly, paediatric thyroid biopsies should also be performed by radiologists (or surgeons) with a high level of proficiency in such procedures, and management should be overseen, or done in consultation, with a paediatric endocrinologist or general paediatrician with experience in managing thyroid nodules.

Ultrasound features of concern for thyroid nodules may include one or more of the following: solid, hypoechogenic, increased vascularity, microcalcifications, indistinct margins, ‘taller‐than‐wider’ morphology, abnormal background gland echotexture and enlarged regional lymph nodes.^5^, ^19^ In adults, Thyroid Imaging Reporting and Data Systems (TI‐RADS) are widely accepted and used to stratify the risk of cancer in thyroid nodules. Their use has led to a significant reduction in unnecessary biopsies in adults.^20^ However, these systems are not yet validated in the paediatric population. A recent study concluded that the American TI‐RADS criteria were insufficient in the evaluation of thyroid nodules in those <18 years; with the use of these criteria up to 22% of cancers cases would not have been referred for biopsy at initial presentation and 10% of cases not been followed up at all.^21^

### Indications for fine needle aspiration

A decision on whether to perform an FNAB is based on clinical and radiological features. In both adults and children, larger nodule size is associated with an increased malignancy risk.^5^ However, absolute size is not as helpful in children, where the decision to FNAB is based on ultrasound features, interval growth, and the presence of cervical lymphadenopathy.^1^, ^7^, ^22^ Given the absence of definitive criteria, the authors recommend that the decision to FNAB be undertaken after multidisciplinary discussion involving experienced paediatric radiologists, surgeons and endocrinologists.

### How should a nodule declared benign on FNAB be followed up?

**Fig. 2:**
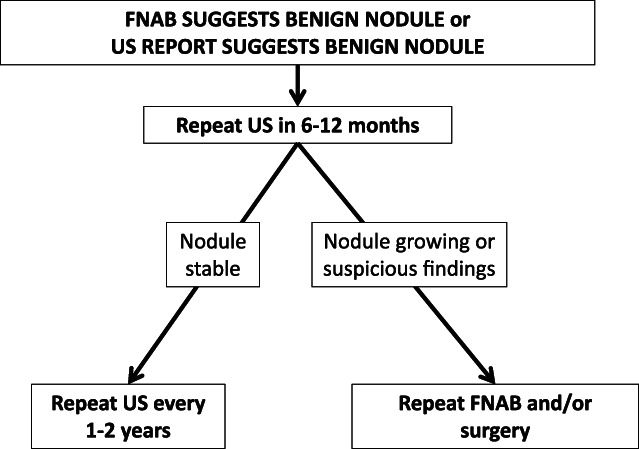
Follow‐up of benign thyroid nodules in children.

ATA guidelines^1^ suggest serial ultrasound follow‐up with repeat FNAB if the nodule increases in size. These guidelines do not clarify how long this follow‐up should continue for, nor do they give guidance for nodules without suspicious features on ultrasound. The authors' suggested approach is detailed in Figure 2.

### How should a nodule declared indeterminate be followed up?

ATA guidelines recommend definitive surgery over repeat FNAB in those with indeterminate cytology due to the higher malignancy rates in children.^1^ However, more recent data have suggested follow‐up should depend on the specific classification within these indeterminate groups, with some able to be followed safely with repeat FNAB.^17^, ^22^, ^23^ The ATA guidelines recommended definitive surgery is total thyroidectomy. However, diagnostic hemithyroidectomy is gaining popularity in response to recent data suggesting a lower incidence of malignancy in indeterminant nodules than first thought.^24^

FNABs should not be performed within 3 months of each other, in order to avoid the reactive cellular atypia that would follow the previous FNAB.^25^

## Thyroid Cancer in Children and Adolescents

Thyroid cancer is the most common paediatric endocrine malignancy representing 1.5% of all childhood cancers.^26^ Its incidence has increased over the past 30 years.^27^, ^28^ The incidence of paediatric thyroid cancer in the USA, based on the Surveillance, Epidemiology and End Results (SEER) registry was 5.9 cases per 1 000 000 population between 2007 and 2012,^28^, ^29^ with the incidence increasing with age. In younger children, boys and girls are at similar risk, but after puberty there is a higher incidence in girls (5:1 female to male predominance).^28^

Paediatric thyroid cancer differs significantly from that seen in adults. Children often present with more advanced disease but have a better response to treatment and prognosis. At diagnosis, children have higher rates of multifocal disease (80–90% compared with 40–50%), lymph node extension (40–90% vs. 20–50%) and distant metastases (20–30% vs. 2%).^1^ Despite this, thyroid cancer in children has better long‐term prognosis with a 10‐year survival rate of up to 98%.^26^ Paediatric thyroid cancers also differ genetically, with some forms of thyroid cancer more frequently seen in the paediatric age group.^1^, ^30^

Most paediatric thyroid cancers are well differentiated thyroid cancers, with anaplastic thyroid cancer being very rare. Papillary thyroid carcinoma (PTC) represents the majority (60%) of cases. Other types of thyroid cancer seen in children include the follicular variant of PTC (23%), follicular thyroid carcinoma (FTC) (10%) and medullary thyroid cancer (MTC) (5%).^28^ The diffuse sclerosing variant of PTC, presents with non‐nodular, diffuse infiltration of the thyroid with microcalcifications throughout, and is more common in children and young adults than older adults.^5^

Risk factors for PTC include iodine deficiency, prior radiation exposure to the thyroid gland and familial cancer syndromes (Cowden, DICER1, APC‐associated polyposis and Carney complex). The differences in the clinical presentation and outcome in paediatric compared with adult thyroid cancer patients may be partly explained by differences in genetic profiles. In children with PTC, gene rearrangements are more common than point mutations in proto‐oncogenes.^31^ RET proto‐oncogene rearrangement mutations are more common in children than in adults (47–65% vs. 3–34%).^26^ In contrast, sporadic BRAF proto‐oncogene point mutations, which are associated with a poorer prognosis, are uncommon in children compared with adults (3–6% vs. 40–70%).^26^ Paediatric follicular thyroid carcinoma (FTC) is usually minimally invasive (90%), rather than widely invasive.^32^, ^33^ Compared to PTC, which is frequently multifocal involving lymph nodes, FTC is usually unifocal, and is unlikely to spread to regional lymph nodes, but is more prone to haematogenous metastases to the bones and lungs.^1^, ^34^ Minimally invasive FTC is managed surgically, usually with total thyroidectomy. Some tumours may be manageable with hemi‐thyroidectomy alone. Invasive FTC or larger tumours (>4 cm) are managed with total thyroidectomy and post‐operative RAI.^1^, ^35^, ^36^ As with PTC, post‐treatment surveillance includes measurement of Tg, serial examination and ultrasound.^1^ Another important consideration in FTC is genetic counselling and testing for germline phosphatase and tensin homologue (PTEN) mutations.^1^ Medullary thyroid carcinoma represents just 5% of thyroid cancers in children, with 95% inherited as part of MEN2 or the related variant familial MTC. These syndromes are sometimes diagnosed prior to the onset of MTC and may prompt early prophylactic thyroidectomy.^37^ The timing of prophylactic thyroidectomy is dependent on risk stratification which is in turn dependent on identifying the relevant variant of the associated RET mutation. A diagnosis of MTC is supported by an elevated serum calcitonin. Calcitonin levels can be used in post‐treatment surveillance for residual or recurrent disease.^37^ As with PTC, standard treatment is with total thyroidectomy. Compared to PTC, there is a greater role for central nodal dissection at the time of thyroidectomy to assess for, and to treat nodal involvement. This is because RAI does not play a role in the management of MTC and cannot be used to treat microscopic nodal disease. Lateral cervical lymph node dissection should generally be reserved for biopsy‐proven nodal metastases.^46^ Though RAI is not effective in MTC,^38^ Vendetanib, a tyrosine kinase inhibitor targeting the RET oncogene and vascular endothelial growth factor receptors, has been shown to be effective in the adjuvant setting.^37^, ^39^ In the future, targeted therapies may decrease the dependence on surgery for the management of MTC.^26^

### Management of Paediatric Thyroid Cancer 

Paediatric thyroid cancer management should be undertaken by a multidisciplinary team in a specialist centre with access to paediatric endocrinology, nuclear medicine, paediatric oncology, paediatric radiology, paediatric anaesthesia, high‐volume thyroid surgery and paediatric intensive care.^1^

### Staging and classification

The American Joint Committee on Cancer (AJCC) Tumour‐Node‐Metastasis (TNM) classification^40^ classifies all thyroid cancer patients under 55 years as stage I (no distant metastases) or stage II (distant metastases). Within stage I, there is a wide spectrum of disease. The most recent ATA guidelines^1^ proposed a new staging system for paediatric PTC where ‘low‐risk’ describes disease confined to the thyroid gland with N0 or NX or incidental N1a metastases. The ‘low‐risk’ group is at the lowest risk of distant metastases but may still be at risk of residual cervical disease, especially if central neck dissection is not performed. ATA ‘intermediate‐risk’ describes those with extensive N1a or minimally invasive N1b disease, who are also at low risk of distant metastases but have a high risk of persistent cervical disease. Locally invasive disease (T4) or regionally extensive disease (extensive N1b) with or without metastases is classified at ‘high‐risk’.

### Surgical management of PTC in children

Surgical management of PTC in children differs significantly from that in adults. Total thyroidectomy (rather than hemi‐thyroidectomy) is recommended for children with PTC due to the high risk of bilateral/multifocal disease and recurrence.^1^, ^30^ Research in children with PTC demonstrates an increased risk of recurrence associated with ‘sub‐total’ thyroidectomy,^1^, ^41^, ^42^ and one study showed that total thyroidectomy reduced recurrence rates from 35 to 6% over a 40‐year follow‐up period.^41^ Additional benefits of total thyroidectomy include allowing the use of iodine 131 to detect and treat residual disease, as well as serum thyroglobulin measurement as surveillance for recurrent disease.

Ipsilateral central neck dissection should be considered in all cases of paediatric PTC. Central neck dissection is absolutely indicated when central nodal metastases are suspected radiologically or have been biopsy‐proven.^1^, ^30^ Lateral neck dissection is indicated for biopsy‐proven lateral lymph node metastases.^43^

Complications of thyroidectomy and/or central neck dissection include transient, or rarely permanent, hypoparathyroidism, and recurrent laryngeal nerve damage. Complications of lateral neck dissection include spinal accessory nerve injury, Horner's syndrome from damage to the sympathetic chain, sensory deficits in the distribution of the cutaneous supply of the cervical plexus, and chyle leaks. To minimise complications, thyroid surgery should be performed by a high‐volume surgeon (performing more than 30 cervical endocrine procedures per year).^1^, ^19^

### Radioactive iodine therapy

Adjuvant radioactive iodine (RAI) ablation of the thyroid bed is recommended for children with intermediate‐ or high‐risk disease. RAI confers an improved 10‐year disease‐free survival and a reduction in the rate of local recurrence.^1^, ^42^, ^44^

Despite limited evidence in children, there is a concern that RAI may potentially predispose them to the development of treatment‐associated malignancies in the salivary glands, colon, rectum, soft tissue and/or bone.^1^ RAI also carries a risk of mild myelosuppression, sialadenitis, transient impairment of gonadal function (but no evidence of reduced long‐term fertility), nausea and vomiting.^1^, ^45^ In addition, pulmonary fibrosis in those with pulmonary metastases receiving RAI is a significant dose‐dependent risk.^46^

### Post‐operative monitoring and management

A diagnostic iodine scan and/or TSH‐stimulated serum thyroglobulin (Tg) should be performed 6–12 weeks post‐operatively and may be used in post‐operative staging. Both require a TSH level of >30 mU/L. This is achieved by withdrawing thyroxine therapy for 2–3 weeks or with the use of recombinant human TSH.^1^, ^26^ For those with low‐risk disease, ATA recommends that the measurement of TSH‐stimulated Tg alone may be used to assess patients for the presence of residual disease, with confirmation on nuclear imaging indicated in intermediate‐ or high‐risk PTC.^1^

Post‐thyroidectomy management includes a TSH‐suppressing dose of thyroxine to reduce stimulation of residual thyroid tissue.^26^, ^46^ Long‐term monitoring includes 6–12 monthly ultrasounds and measurement of Tg.^26^ Anti‐thyroglobulin antibodies (TgAb) can complicate interpretation of Tg levels. However, serial measurement of TgAb is an alternative surveillance method; a rising TgAb titre suggests disease recurrence.^47^, ^48^ Remission is defined as the combination of an undetectable Tg, negative TgAb, no evidence of cervical disease on thyroid and lateral cervical ultrasound and a negative thyroid scan.^1^ Patients should be followed up long‐term, although there is no consensus on total duration of follow‐up required. While the majority of thyroid cancer recurrences occur within 5 years of initial therapy, older studies have shown late recurrences occurring after more than 20 years.^46^

## Conclusion

Thyroid nodules in children pose a significant diagnostic challenge. Although thyroid nodules are relatively uncommon in children as compared to adults, they require careful assessment due to an increased malignancy risk. Although thyroid cancer in children usually presents with advanced disease, prognosis is usually favourable. Thyroid cancer management in children differs from that of adults and requires a multidisciplinary team, including a paediatric endocrinologist, high‐volume thyroid surgeon, nuclear medicine physician, paediatric radiologist, paediatric oncologist and a psychosocial support team.
